# Case Report: Neurological immune-related adverse events with anti-PD-1 based immunotherapy in cervical cancer

**DOI:** 10.3389/fonc.2026.1796299

**Published:** 2026-05-14

**Authors:** Gaia Passarella, Sofia Vella, Erminia Ferrario, Nicoletta Provinciali, Diego Luigi Cortinovis, Stefania Canova

**Affiliations:** 1 Medical Oncology Unit, Fondazione IRCCS San Gerardo dei Tintori, Monza, Italy; 2 Medicine and Surgery Department, University of Brescia, Brescia, Italy; 3 Medicine and Surgery Department, University of Milano Bicocca, Monza, Italy

**Keywords:** cemiplimab, cervical cancer, immune checkpoint inhibitors, immune-related adverse events, myelitis, myositis

## Abstract

Despite recent advances in screening and primary prevention, cervical cancer (CC) still represents the fourth leading cancer in terms of incidence and mortality among women. Immune checkpoint inhibitors (ICIs) have gradually emerged as a promising treatment option in the management of CC. However, their use is associated with immune-related adverse events (irAEs), which may occur up to several months after starting or ending therapy. Although irAEs can potentially affect any organs or systems, some sites, such as the nervous system, are seldom involved. We report on the case of a 54-year-old patient with HPV-related, recurrent and metastatic CC treated with cemiplimab. Following an initial favourable partial response, the treatment had to be interrupted due to the onset of immune-related pneumonitis. Subsequently, during steroid tapering, the patient developed myositis and myelitis requiring hospitalization. Further investigations including cerebrospinal fluid analysis, serologic testing, virological and microbiological studies, and radiological imaging excluded infectious, malignant and primary inflammatory neurological aetiologies, supporting the hypothesis of a delayed irAE. Despite immunosuppressive treatment with high-dose steroids and a course of plasmapheresis, the patient gained only partial clinical improvement, requiring a rehabilitation treatment in a specialised facility. The widespread use of immunotherapy in advanced-stage malignancies is expected to increase the incidence of irAEs, including rare and severe forms such as neurological events and those with delayed onset. Consequentially, structured and prolonged follow-up is essential for early identification and effective management of ICIs toxicities.

## Introduction

Cervical cancer (CC) ranks as the fourth most common cancer and leading cause of cancer-related death among women worldwide with an incidence of 662, 044 cases and mortality of 348, 079 women in 2022 (1). CC has a significant impact in developing countries due to exposure to risk factors and lack of screening programs as vaccination against high-risk types of human papillomavirus (HPV) and PAP test/acetic visual inspection (VIA) (2). In 2022, in Italy there were 2, 500 new cases of CC, with a prevalence of 51, 100 cases (3).

Cervical cancer staging and treatment follow guidelines set by the International Federation of Gynaecology and Obstetrics (FIGO), updated in 2018 and European Society of Gynaecological Oncology (ESGO) guidelines updated in 2023 (4, 5).

At diagnosis, about 37% of patients have locally advanced disease (FIGO IB2-IVA) and one third of patients at this stage experiences disease recurrence following chemoradiation (6, 7). Around 10% of newly diagnosed CC patients present with distant metastases (FIGO stage IVB) and their prognosis is poor (6, 8). Based on these findings, a significant number of CC patients should be considered for systemic therapeutic approaches.

First-line treatment for patients with recurrent or metastatic CC includes chemotherapy combined with immunotherapy, with or without bevacizumab (a vascular endothelial growth factor inhibitor). In October 2021 and April 2022 respectively, based on the results of the KEYNOTE-826 trial, the Food and Drug Administration (FDA) and the European Medicines Agency (EMA) approved the use of pembrolizumab (a humanised monoclonal anti-programmed cell death protein-1 (PD-1) antibody) in combination with platinum-based chemotherapy, with or without bevacizumab, for patients with programmed death-ligand 1 (PD-L1) – positive recurrent or metastatic disease who were not eligible for curative treatment. For PD-L1–negative tumours, chemotherapy combined with bevacizumab remains the standard of care (9, 10).

Treatment with cemiplimab, an anti PD-1 agent, should be considered, regardless of PD-L1 expression, for patients with recurrent or metastatic CC with disease progression following first-line platinum-based chemotherapy and naïve for immunotherapy (4). The phase III EMPOWER-Cervical 1 trial demonstrated improved overall survival with cemiplimab as second-line therapy compared to single-agent chemotherapy, especially in patients with PD-L1 expression ≥1% (9, 11). EMA approved cemiplimab in this setting in November 2022 and Italian Medicines Agency (AIFA) in July 2024 (12, 13).

For patients with disease progression following chemotherapy and immunotherapy, antibody–drug conjugates offer a potential treatment option. FDA has approved trastuzumab deruxtecan, a humanised anti-HER2 (Human Epidermal Growth Factor Receptor 2) monoclonal antibody linked to a topoisomerase I inhibitor for HER2 - expressing IHC (immunohistochemistry) 3+ solid tumours of any type. In addition, tisotumab vedotin (an anti-tissue factor monoclonal antibody linked to the tubulin inhibitor monomethyl auristatin E) has been approved for metastatic CC patients after chemotherapy as second- and third-line treatment (9, 14–17).

This case highlights the complex and unpredictable nature of irAEs, particularly those occurring during steroid tapering, and the importance of recognizing and managing delayed immune-related toxicities, especially in the context of CC, for which literature is currently limited.

## Clinical case

**Figure 1:**
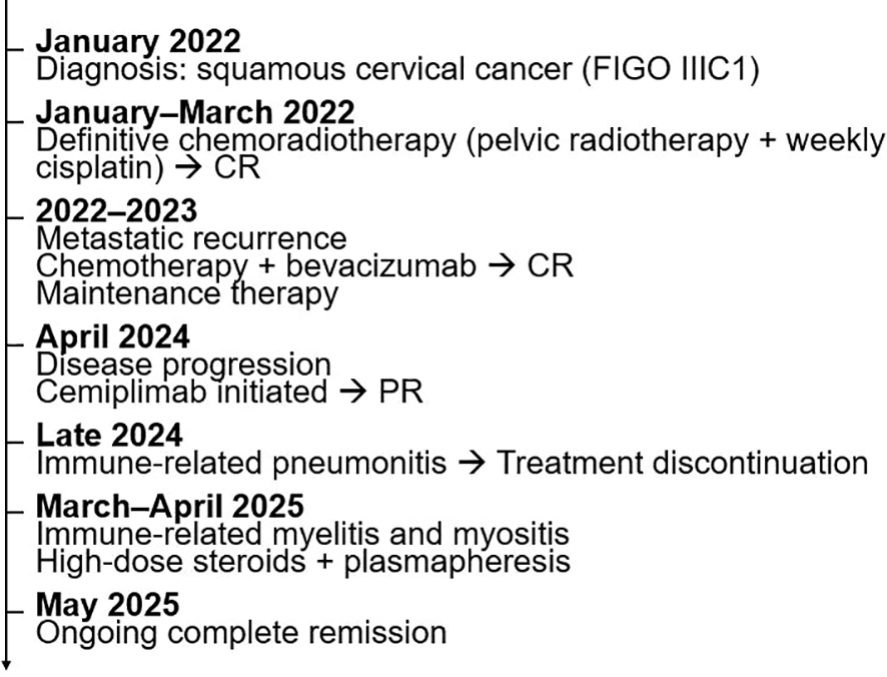
Clinical timeline of the patient. CR, Complete Response; PR, Partial Response.

A 54-year-old female with a negative family history for neoplastic diseases was diagnosed in January 2022 with HPV-related grade 2 squamous CC with suspected radiologically iliac lymphadenopathies (stage IIIC1 according to FIGO 2018). She underwent radiotherapy on positive lymph nodes and uterus in combination with weekly cisplatin chemotherapy, followed by an external beam boost to the uterus, achieving a complete response on the subsequent monitoring CT scan (**Figure 1**).

Approximately 7 months later, fine-needle aspiration cytology of a PET-avid hilar–mediastinal region confirmed metastatic recurrence of the primary CC. A first line treatment with carboplatin and paclitaxel in combination with bevacizumab was started with a complete response on PET scan after 6 cycles, thus maintenance therapy with anti-angiogenic alone was continued.

In April 2024, a PET/CT scan showed disease progression in both cervix and mediastinal lymph nodes. PD-L1 expression was tested on hilar lymph node with a combined positive score (CPS) of 40, so that we were allowed to start immunotherapy with cemiplimab through a compassionate use program. After 3 cycles of therapy the patient obtained a partial response of the mediastinal lymph nodes and stability of the cervical lesion.

After 7 cycles, the patient developed dyspnoea and dry cough. CT imaging showed diffuse alveolitic alterations in almost all lung fields with no evidence of suspicious metastatic lesions, compatible with grade 2 immunotherapy-related pneumonitis according to multidisciplinary team. Laboratory inflammatory markers were negative.

According to guidelines, cemiplimab treatment was suspended and steroid therapy with prednisone 50 mg/day was immediately started, with close outpatient monitoring (18).

After a few days, the patient was hospitalised due to worsening symptoms with acute respiratory failure. Bronchoscopy with bronchoalveolar lavage was performed with negative microbiological analysis. Intravenous steroid therapy with methylprednisolone 40 mg bid was administered along with ventilatory support with Venturi mask at 50%. An antibiotic prophylaxis with trimethoprim/sulfamethoxazole and levofloxacin was prescribed. Clinical and imaging improvement was recorded after 1 week of treatment.

Upon discharge, the patient received a corticosteroid tapering regimen, starting with prednisone 50 mg/day for two weeks, reduced to 37.5 mg/day for the following two weeks, and finally to 25 mg/day until the next scheduled instrumental follow-up.

Surveillance was scheduled every two months with chest-abdominal CT scans showing persistent and stable interstitial lung disease. In March 2025, during steroid tapering (at the time prednisone 25 mg/day), the patient was admitted in the emergency room for right lower limb hyposthenia and persistent pain. Clinical examination revealed anaesthesia of the right lower limb, and dysesthesia of the right distal extremity. Laboratory tests showed progressive increase of creatine phospokinase (CPK) to a peak of 5817 U/L and elevated C-reactive protein (CRP) with a peak of 50.9 mg/L.

**Figure 2:**
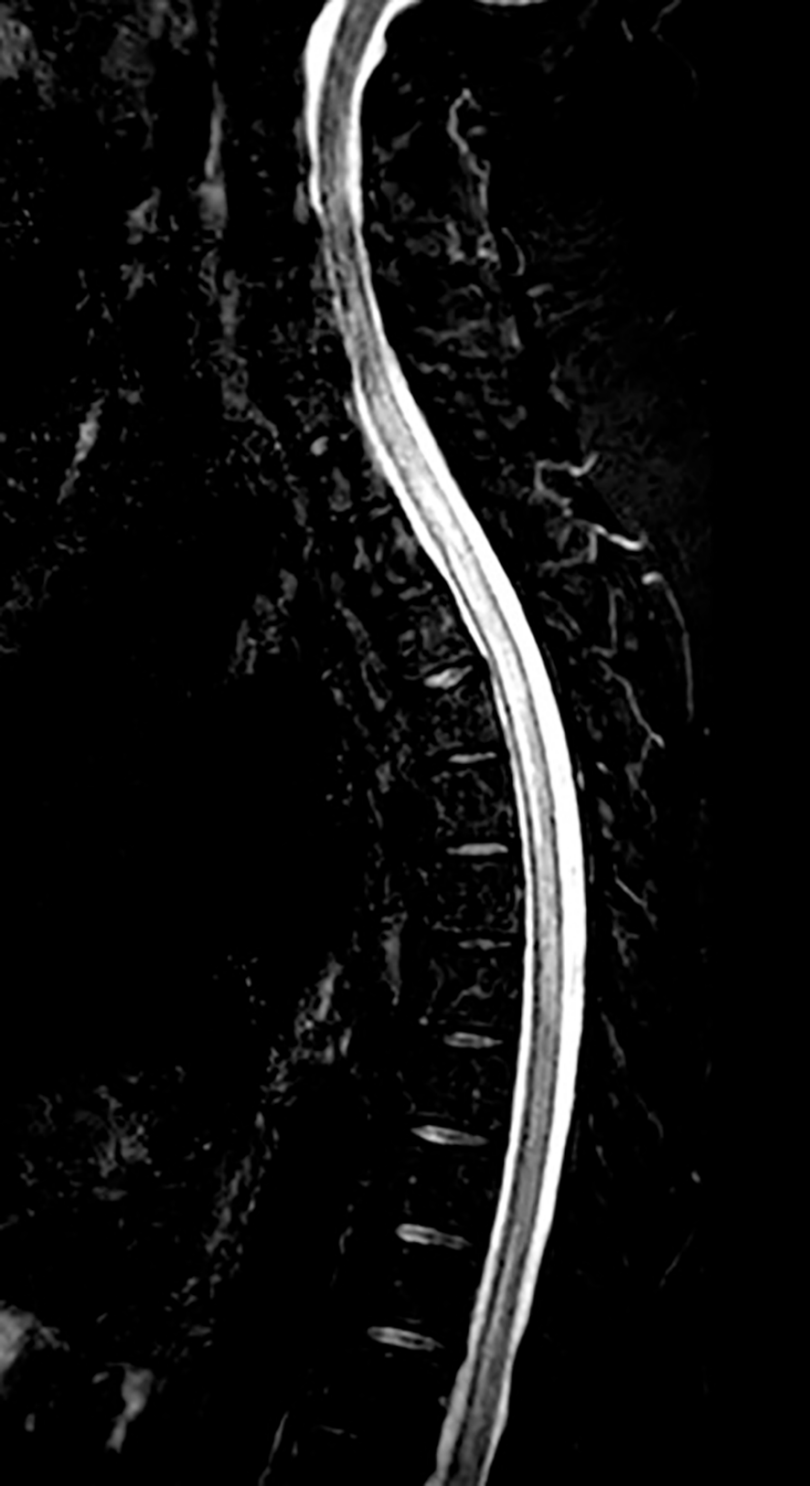
Myelitis extending from C7 to T7 evidenced by spinal MRI - T2-weighted sequences STIR (short tau inversion recovery) fat suppression.

Lumbar spine X-ray and thoraco-abdominal CT were negative for acute findings but showed stable interstitial lung lesions, consistent with post-immunotherapy pneumonitis. Brain CT with contrast was negative for both oncologic and vascular events. The patient received analgesic therapy with pregabalin (a gabapentinoid anticonvulsant) and intravenous hydration in the Emergency Room; she was subsequently admitted to the oncology department and later transferred to the neurology unit for further diagnostic investigations. Spinal MRI revealed findings consistent with myelitis extending from C7 to T7 (**Figure 2**). A lumbar puncture was performed. The cerebrospinal fluid (CSF) appeared clear and colourless, bacterioscopic examination showed only rare monocytes (less than one per microliter). CSF analysis revealed a mild protein elevation with a normal cell count. No free kappa or lambda light chains were detected in the CSF, and the search for oligoclonal IgG bands was negative. The Link index was 0.60. CSF analysis showed an immunoglobulin G level of 56.2 mg/L (normal range 0.0–34.0 mg/L) and an albumin level of 474.1 mg/L (normal range 110.0–340.0 mg/L). Serum free kappa and lambda light chains, as well as serum immunoglobulin G levels, were within the laboratory reference ranges. The presence of paraneoplastic antibodies in the serum and CSF was not evaluated. Virological testing of the CSF was negative, including assays for human herpesvirus 1–2–3–6–8 DNA, parvovirus B19 DNA, enterovirus RNA, adenovirus DNA, cytomegalovirus DNA, and Epstein–Barr virus DNA. Bacteriological and mycological investigations were also negative with nucleic acid testing of the CSF showing no evidence of Escherichia coli K1, Haemophilus influenzae, Listeria monocytogenes, Neisseria meningitidis, Streptococcus agalactiae/pneumoniae, and Cryptococcus neoformans/gattii. Consequently, the infectious aetiology was excluded. Thereby, high-dose intravenous methylprednisolone of 1 g/day was initiated.

**Figure 3:**
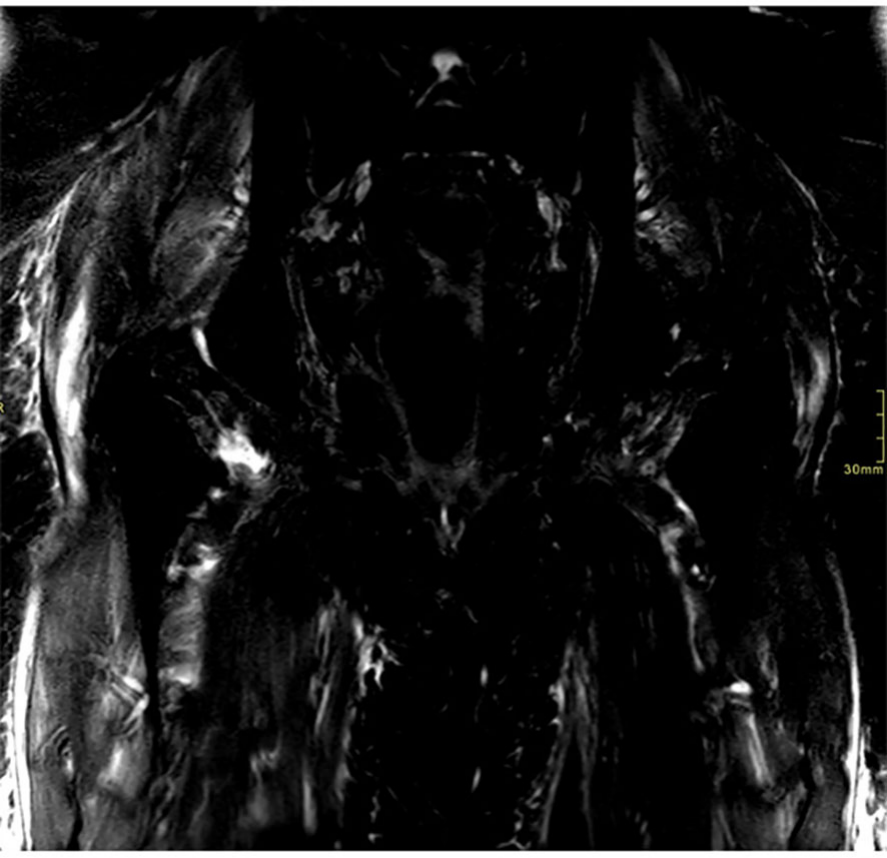
MRI of the pelvis and femurs with evidence of bilateral myositis - T2-weighted sequences STIR fat suppression.

Autoimmune myositis panel and electromyography were performed, which suggested a myositis process. An MRI of the pelvis and femurs (**Figure 3**) showed findings consistent with bilateral myositis, more prominent on the right side while thoracic-abdominal CT scan evidenced complete remission of cancer. Transthoracic echocardiogram ruled out a myocardial involvement.

After 5 days on intravenous steroid treatment with clinical and laboratory improvement, the therapy was switched to oral prednisone at a dose of 1 mg/kg/day. Due to the administration of high doses of steroid, laboratory investigations of immune system and active opportunist infection were performed, showing low level of total lymphocytes and CD4+ T-cell counts (210/mm³); EBV-DNA was detected at 106 copies/mL combined with serology consistent with a past infection. Infectious disease consultation recommended to continue prophylactic trimethoprim-sulfamethoxazole (800/160 mg, one tablet three times per week). Weekly monitoring of EBV-DNA levels was advised and so far has shown negative results.

**Figure 4:**
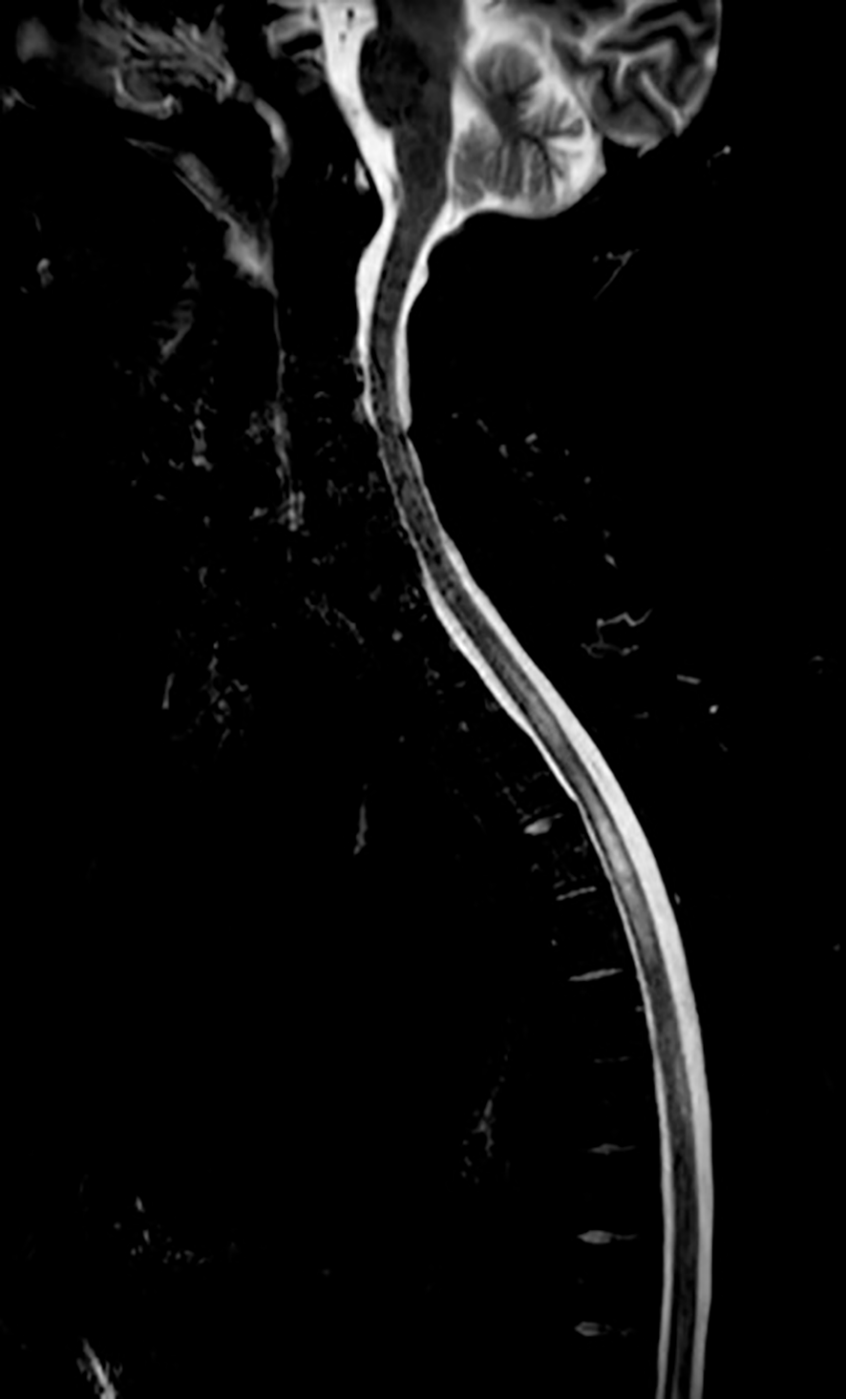
Spinal MRI performed approximately five weeks after the first imaging (refer to **Figure 2**) - T2-weighted sequences STIR fat suppression.

Spinal, pelvic, and femoral MRI performed two weeks after the previous examinations showed a reduction in oedema of the muscle and subcutaneous adipose tissues, a modest decrease in spinal cord swelling, but more evident leptomeningeal enhancement along the margins of the conus medullaris and the roots of the cauda equina (**Figure 4**). To exclude leptomeningeal carcinomatosis, a diagnostic lumbar puncture was performed showing no evidence of malignant tumour cells.

Due to limited clinical response to steroid therapy, a cycle of plasmapheresis with five sessions on alternate days was administered in combination with physiotherapy resulting in an improvement of the clinical conditions. Approximately 20 days later, a new spinal MRI revealed further slight improvement of the neuroradiological findings, both at the intramedullary level in the dorsal cord and in the leptomeninges of the conus medullaris and cauda equina (**Figure 4**).

A new EMG also showed improvement of axonal motor polyneuropathy. Therefore, discharge from hospital and transfer to a rehabilitation facility were allowed.

The long-term hospitalisation was marked by several complications: the patient developed both recurrent urinary infections and a thrombosis in the right common femoral and gastrocnemius veins of the left calf, which required anticoagulant treatment. Furthermore, the patient developed a depressed mood, likely related to her near-complete dependence in activities of daily living; therefore, psychological support was initiated on medical recommendation and was well received.

The cancer remained in complete remission at the last CT scan performed in May 2025.

## Discussion

The increasing use of immune checkpoint inhibitors (ICIs) for the treatment of gynaecological cancers requires early recognition and prompt management of irAEs, which can sometimes be severe or even life-threatening if not properly addressed. Therefore, clinical evaluation must always include the assessment of immune-mediated toxicity, especially in the presence of non-specific presentations.

Toxicities vary by treatment regimens: grade ≥3 irAEs occurring in 7–28% of patients on anti-PD-1/PD-L1 monotherapy, 48–59% with PD-1/CTLA-4 (cytotoxic T-lymphocyte-associated protein 4) combinations, and around 19% with PD-1/anti-LAG-3 (lymphocyte-activation gene 3) therapy (19). They predominantly involve the gastrointestinal tract, endocrine glands, skin, and liver; nervous, cardiovascular, pulmonary, musculoskeletal, and hematologic systems are less commonly affected (20). According to Dubey et al., neurological irAEs occur in 1%–3% of patients treated with ICIs (21), likely underestimated given the under-reporting of AEs whose symptoms might be mild or non-specific (22). They encompass a wide range of possible manifestations including myositis, peripheral and cranial neuropathies, meningitis, and encephalitis. Though rare, these events represent a significant clinical concern due to their potential severity and the risk of life-threatening complications (e.g. myocardial or bulbar involvement) (18, 23). Mikami et al. conducted a retrospective observational study about neuroimmunological AEs associated with ICIs using the FAERS (United States Food and Drug Administration Adverse Event Reporting System) data collected between January 2014 and December 2019. They observed that neuropathy was the most common neurologic irAE (2.7%) among all the 3, 619 patients that experienced neurologic irAEs, followed by hypophysitis/hypopituitarism (1.8%), myositis (1.1%), and encephalitis/myelitis (0.9%). They noted that ICI combination therapy increases the risk of neurological irAEs compared to ICI monotherapy, especially in older patients (24).

Systemic corticosteroids remain the first-line treatment for irAEs (19). According to the ESMO (European Society for Medical Oncology) guidelines, immunotherapy can be continued in combination with close monitoring with grade 1 symptoms. For grade 2 irAEs, immunotherapy should be discontinued, and oral or intravenous l methylprednisolone should be started at dosage of 0.5 mg/kg/day. For grade 3 and 4 irAEs, an increased dose of methylprednisolone (1–2 mg/kg/day) is necessary and, for life threatening cases, methylprednisolone bolus at a dose of 1 g/day for 3–5 days should be considered (18). Targeted immunomodulatory therapies have shown efficacy in steroid-refractory cases: anti–TNF-α (tumour necrosis factor-α) agents (i.e. infliximab), mycophenolate mofetil, IL-6 inhibitors (i.e. tocilizumab), and JAK (janus kinase) inhibitors (i.e. tofacitinib). In refractory cases, intravenous immunoglobulin (2 g/kg/day within 3–5 days) and plasmapheresis, may be necessary (18, 19). Corticosteroids tapering should be conducted over a period of 4–8 weeks depending on the severity of the symptoms, extendable up to six months if symptoms recur or worsen during the reduction of dosage (18).

Symptoms may persist, recur or even worsen during corticosteroid therapy, which highlights the importance of defining and better understanding long-term or chronic forms of toxicity. The Society for Immunotherapy of Cancer (SITC) defines chronic irAEs as those “persisting beyond three months of ICI discontinuation” and it classifies them as either inactive or active: the former resulting from permanent organ damage, the latter from ongoing inflammation (25). Rossi et al. observed that chronic active irAEs primarily occur in patients who require ongoing immunosuppression or experience relapse during corticosteroid tapering; their etiopathogenetic model suggests that immune checkpoint blockade may unmask a latent and clinically silent autoimmune process, potentially driven by individual susceptibility or tumour-related antigenic cross-reactivity (23).

In our case, there was not a continuous and chronic symptomatology, but rather the involvement of two distinct systems at different times. The first irAE was an immunotherapy-related pneumonia, developed five months after the initiation of cemiplimab. Subsequently, a neurological irAE emerged approximately five months after the discontinuation of immunotherapy, during the tapering of corticosteroid therapy initiated after the first event. This temporal correlation may suggest the presence of an underlying immune reactivity that had been kept under control by the ongoing steroid treatment until its reduction. CSF analysis showed a mild increase in protein levels with abnormal cell count; the elevated albumin concentration, together with the normal Link index and the absence of oligoclonal IgG bands, suggests blood–brain barrier dysfunction rather than intrathecal immunoglobulin synthesis. This CSF profile may be observed in immune-mediated, toxic, or paraneoplastic conditions. In this case paraneoplastic antibodies were not assessed (26), as they are not routinely included in the standard diagnostic work-up. Cervical cancer is rarely associated with paraneoplastic neurological syndromes (PNS), with only sporadic cases reported (27–29), in contrast to tumours such as small cell lung cancer, breast cancer, ovarian cancer, and lymphomas. However, ICI can trigger specific neurological syndromes (e.g., Ma2- or Hu-related), although many cases remain seronegative at the screening. (30).

Microbiological and virological testing was negative, making an infectious cause unlikely. Radiological assessment showed stable oncological disease, excluding a direct neoplastic cause.

Although the majority of irAEs typically occur within the first three months following the initiation of ICI therapy, it is well established that they can also arise later during treatment or even several months after its conclusion (31). In our case, the events observed do not meet the definition of delayed irAEs, which are generally defined as those occurring more than one year after the initiation of ICI therapy (32, 33). However, as proposed by Couey et al., the episode of myositis may be classified as a delayed immune-related event (DIRE), since it represents a newly emergent irAE occurring 90 days or more after the discontinuation of immunotherapy (34).

Therefore, there is no standardised temporal classification for irAEs. This is mainly due to the limited data on late onset irAEs from clinical trials. Indeed, AEs characteristics, analysis and methods used to assess ICI toxicity in cancer clinical trial publications were poorly reported (35–37). A systematic review by Chen et al. brought attention to methodological limitations in the assessment of irAEs: short follow-up periods (some protocols excluded the reporting of AEs occurring more than 30 days after the last dose), the occasional lack of specification regarding follow-up duration and the omission of irAEs reporting following the initiation of subsequent anticancer therapies (34, 37). Given that irAEs can persist or even worsen after discontinuation of ICIs, adequate follow-up is essential to assess their evolution. IrAEs may not be fully reversible, and their evaluation becomes more challenging if patients initiate subsequent therapies. The complexity of establishing causality may also contribute to the frequent lack of reporting (37).

Focusing on cemiplimab and cervical cancer, the EMPOWER-Cervical 01 trial included post-treatment assessments only at a first follow-up visit 30 days after the last dose and a second final assessment at 90 days. Therefore, the follow-up period was relatively short with a significant risk of underestimation of late irAEs. The study reported that any grade-irAEs occurred in 15.7% of patients treated with cemiplimab with grade ≥3 events in 5.3%. Pneumonitis was observed in 1.3% of patients, while no specific data were available regarding immune-related neurological events (11, 38).

## Conclusions

Given the growing use of immunotherapy in clinical practice, a parallel increase in the incidence of related adverse events is to be expected, including but not limited to the most uncommon ones. These irAEs may sometimes be partially irreversible, even life-threatening, and they can persist or even worsen after discontinuation of ICIs. To our knowledge, this is the first case report of neurological toxicity associated with cemiplimab in cervical cancer. Moreover, the case raises challenging questions about current time-based definitions of irAEs, which may not adequately capture the full spectrum of their clinical manifestations. Therefore, it is necessary to establish adequate follow-up programs for patients undergoing immunotherapy aimed at enabling early detection of potential toxicities and thus optimise therapeutic management to limit the risk of severe or irreversible complications. Considering the possible late onset of immune-related toxicities, surveillance should be extended over time, especially during the tapering of immunosuppressive therapy, even after the end of the oncological treatment.

## Data Availability

The original contributions presented in the study are included in the article/supplementary material. Further inquiries can be directed to the corresponding author.
